# Willingness to participate in HIV research at the end of life (EOL)

**DOI:** 10.1371/journal.pone.0199670

**Published:** 2018-07-23

**Authors:** Katya Prakash, Sara Gianella, Karine Dubé, Jeff Taylor, GaYoung Lee, Davey M. Smith

**Affiliations:** 1 Division of Infectious Diseases, University of California, San Diego, La Jolla, California, United States of America; 2 University of North Carolina Gillings School of Global Public Health, Chapel Hill, North Carolina, United States of America; 3 Community Advisory Board (CAB) AntiViral Research Center (AVRC) San Diego, San Diego, California, United States of America; 4 Johns Hopkins Bloomberg School of Public Health, Department of Molecular Microbiology, Immunology, Baltimore, Maryland, United States of America; 5 Veterans Affairs San Diego Healthcare System, San Diego, California, United States of America; National Institute of Child Health and Human Development, UNITED STATES

## Abstract

**Introduction:**

Animal models have been vital for scientific discovery but have limitations, especially in infectious disease research. It is essential to develop a means to study these diseases in human models. We hypothesized that altruistic people would willingly participate in research near the end-of-life (EOL), for the benefit of science and to provide one last gift to society.

**Methodology:**

Two surveys were administered to 377 self-reported HIV-negative and 96 HIV-positive individuals. Hypothetical questions assessed their willingness to participate in altruistic research in the last 6 months of life, which might result in a shortened lifespan or physical discomforts. The self-reported HIV-negative group was also asked about willingness to be exposed to infectious pathogens for the sake of research.

**Results:**

Almost all responders expressed willingness to participate in research at the EOL, regardless of HIV-status. The majority of participants were willing to endure physical discomfort for the sake of research. ‘Blood draws’ was identified as the most tolerable physical discomfort (>70% in both groups). In both groups, >60% were willing to shorten their lifespans for the sake of research. A third of the self-reported HIV-negative group expressed willingness to be exposed to at least one infectious agent to participate in EOL research.

**Conclusions:**

Our exploratory study demonstrates that people would welcome the opportunity to participate in altruistic research near the EOL. Such research could greatly impact the way infectious disease research is conducted. This study is limited however by its hypothetical nature. Further research is necessary to confirm this interest in those with terminal illness before any further clinical research effort at the EOL can be performed.

## Introduction

In 1937, “Elixir Sulfanilamide”, which contained diethylene glycol, poisoned over a hundred people [1]. In 1938, the Federal Food, Drug and Cosmetic Act was passed requiring safety testing of drugs on animals before they could be marketed [2]. If all goes well, research can eventually progress to humans. As noted by Richard Klausner, former director of the National Cancer Institute, “The history of cancer research has been a history of curing cancer in the mouse. We have cured mice of cancer for decades—and it simply didn’t work in humans.” Furthermore, a 2006 news release by the U.S. Food and Drug Administration (FDA) stated “Nine out of ten experimental drugs fail in clinical studies because we cannot accurately predict how they will behave in people based on laboratory and animal studies.” This is particularly exemplified in Human Immunodeficiency Virus (HIV) research. When HIV was first isolated, mice, rats and rabbits were used for all experiments, but these animals could not be infected with HIV [3]. Eventually, in 1985 the Simian Immunodeficiency Virus (SIV) was isolated from a chimpanzee [4]. Non-human primates, like chimpanzees and rhesus macaques provide a useful animal model for the development of a wide variety of antiretroviral drug testing [5]. Even primate models have limitations and they differ from humans in achievable levels of viremia, rates of disease progression, resistance patterns, immune responses, amongst others [6]. For example, the Merck adenovirus type 5 (Ad5) trivalent HIV-1 vaccine trial (STEP trial) did not show efficacy in preventing HIV infection or even slowing disease progression [7], despite promising results in various macaque studies [8–10]. In fact, the trial had to be stopped in September 2007 when an independent panel of experts’ review revealed that the incidence of infection was *lower* in placebo recipients who had higher levels of Ad5 immunity compared to vaccine recipients [7]. This serves as an example of how divergent animal models can be from human reality.

To date, cancer research has been at the forefront, actively and successfully enrolling participants in Phase I clinical trials that usually harbor considerable risk to the individual [11]. Although there is always hope for tumor response and regression, that is not the primary objective of these trials. The primary objective is to understand pharmacokinetics and drug toxicity [12–15] so investigational interventions can be advanced for further testing.

Researchers have found that some individuals with terminal cancer are willing to participate in research at the End of Life (EOL), even if it had no chance of helping their underlying illness [16]. No such research has yet been conducted concerning infectious disease [17]. Many people are naturally altruistic, and perhaps would take part in a research study that may not have any benefit to them, but may benefit their friends, family or mankind. This altruism may be more acute near the EOL, because it could serve as a last meaningful contribution to society-at-large. Providing those who are terminally-ill the opportunity to participate in clinical research could revolutionize the way therapeutics move from the bench-to-clinic by offering a large supply of well-informed, eager and appropriately consented human volunteers [17].

## Methods

### Study design

This study was comprised of two anonymous surveys. The first was administered to individuals 18 years and older who were self-reported HIV-negative, and the second was administered to self-reported individuals living with HIV. We assessed these two study populations to evaluate EOL research attitude in both the general population and a specific population (i.e. people living with HIV).

The survey with HIV-negative respondents was administered over a period of three months. Participants were recruited online through Amazon’s Mechanical Turk (MTurk) (https://www.mturk.com/mturk/welcome) from all over the United States (non-clinical sample). This platform is commonly used to assess the attitude of the general population and has been validated for this purpose [18,19]. There was a $0.50 monetary compensation provided for participation, which is the common compensation for research studies on MTurk platform.

The survey with people living with HIV was administered from the University of California San Diego’s Owen Clinic. All of the UCSD participants were approached in person with the survey on an anonymous website managed by Qualtrics LLC (https://www.qualtrics.com/). If a participant chose to take the survey, he/she answered questions directly on an iPad, blinded to the recruiter. There was no monetary compensation provided to the HIV-positive group. This group was evaluated for insight into whether an underlying infectious condition or chronic disease would impact attitudes towards EOL research. The proposed study was designed to establish a foundational framework for the next steps in HIV clinical research at the EOL. Based on these results, similar surveys could be developed and implemented for people who are terminally ill with and without HIV.

### Surveys

Both surveys gathered demographic data (e.g. age, gender-identity, race/ethnicity, marital status, household income, education, religious affiliation and current perception of health). Details regarding HIV/AIDS status, time of diagnosis, viral load and CD4^+^ counts were collected from the HIV-positive group. Participants were asked a series of questions regarding their willingness and desire to participate in scientific research that would not benefit them directly but could contribute to the advancement of medical research. They were asked what physical discomforts they would be willing to endure, including but not limited to nausea, vomiting, diarrhea or headaches.

The self-reported HIV-negative participants were asked if they would be willing to be exposed to and infected with pathogens like Streptococcus, Malaria, HIV or Hepatitis C near the EOL, for the sole purpose of research. Responders could select as many or as few of the options provided. All participants were asked if they would consider participating in this research if it had the potential of shortening their lifespans further, and if so, by how long.

### Statistical analysis

All statistical analyses were performed using SAS 9.4. Univariate analyses were performed to compare the demographic characteristics between HIV-positive and self-reported negative groups, using Fischer’s Exact Test. We did not test group differences in attitudes about participating in research because the HIV-positive and HIV—negative groups were different in regard to demographics and other characteristics (Table 1).

**Table 1:** Characteristics of HIV negative and positive groups.

		Demographics n (%)		p Value
		HIV- (n = 377)	HIV+ (n = 96)	
Gender	Male	182 (48.3)	84 (88.4)	< .001
	Female	192 (50.9)	6 (6.3)	
	Transgender/Genderqueer	3 (0.8)	5 (5.3)	
Age	18–24 years	61 (16.2)	1 (1.0)	< .001
	25–44 years	217 (57.6)	30 (31.3)	
	45–64 years	85 (22.5)	54 (56.3)	
	65+ years	14 (3.7)	11 (11.5)	
Income	$0 - $25,000	106 (30.0)	67 (70.5)	< .001
	$25,001 - $50,000	105 (29.7)	18 (18.9)	
	$50,001 - $75,000	75 (21.2)	5 (5.3)	
	$75,001 - $100,000	25 (7.1)	3 (3.2)	
	>$100,000	42 (11.9)	2 (2.1)	
Education	< HS or HS/GED	40 (11.3)	24 (25.3)	< .001
	At Least Some College	230 (65.2)	65 (68.4)	
	Masters/Advanced Degree	83 (23.5)	6 (6.3)	
Race/ethnicity	White	271 (77.0)	38 (39.6)	< .001
	Hispanic	23 (6.5)	27 (28.1)	
	Other	58 (16.5)	31 (32.3)	
Marital status	Single	150 (42.3)	55 (57.3)	< .001
	Married, no children	44 (12.4)	12 (12.5)	
	Married, w/ children	84 (23.7)	3 (3.1)	
	Divorced, widowed, or separated	27 (7.6)	12 (12.5)	
	Living w/partner	50 (14.1)	14 (14.6)	
Religion	Not religious	209 (58.9)	45 (47.9)	< .001
	Catholic	39 (11.0)	28 (29.8)	
	Protestant	50 (14.1)	7 (7.4)	
	Other	57 (16.1)	14 (14.9)	
Number of children	No children	228 (65.5)	74 (77.1)	0.085
	1 child	41 (11.8)	4 (4.2)	
	2 children	40 (11.5)	10 (10.4)	
	3+ children	39 (11.2)	8 (8.3)	
Health status	Healthy	318 (91.9)	76 (79.2)	< .001
	Sick, not terminal	27 (7.8)	20 (20.8)	
	Terminally Ill	1 (0.3)	0 (0)	

HS = High School; GED = General Equivalency Diploma. Group differences for all variables were assessed using the Fischer’s Exact Test. Percentages are based on the number of participants who indicated a specific response divided by the number of participants who responded to the item in question.

## Results

### Ethics statement

The study was approved by the Institutional Review Board at the University of California San Diego. All adult participants (age ≥ 18 years) provided written informed consent. No children were included in this study.

### Participant characteristics

#### Self-reported HIV-negative group (non-clinical sample)

Of the 377 eligible participants in the self-reported HIV-negative group, 50.9% (*n* = 192) were female, and 3 identified as transgender. Over half the participants (57.6%) were aged between 25 and 44 years, while 16.2% were between ages 18–24, 22.5% between 45–64 and only 3.7% were over the age of 65. Ethnically, this group identified predominantly as non-Hispanic Caucasian (77.0% *n* = 271). The HIV-negative group was highly educated with 65.2% (*n* = 230) having at least started college and another 23.5% having completed an advanced degree. Further, 40.2% (*n* = 142) reported an annual salary of >$50,000 and 30.0% reported an annual income between $0–25,000. With regards to marital status, half were married or in a relationship (50.1%, *n* = 178). Concerning religion affiliation, 58.9% (*n* = 209) of HIV-negative participants self-identified as “nonreligious”. From the respondents of a practicing faith, 14.1% (*n* = 50) were Protestant, 11.0% (*n* = 39) Catholic and the remaining were practicing Buddhists, Hindus, Muslims, Jews and/or Other. Concerning heath perception, 7.8% of participants reported feeling ‘sick but not terminally-ill’ (Table 1).

#### HIV-positive group

Of 119 individuals in the HIV-positive group, 15 surveys were excluded due to participants’ acquittal mid-administration and another 6 because the individuals reported not being HIV-infected. Of the remaining 96 participants, the majority (88.4%, *n* = 84) were male, 4 identified as transgender and one as genderqueer. Regarding age, over 56.3% responders (*n* = 54) in the HIV-positive group were between the ages of 45–64, 31.3% between the ages of 25–44 years, and only one participant was in the younger age group. Participants were ethnically diverse with 39.6% (*n* = 38) identifying as non-Hispanic Caucasian, 28.1% as Hispanic, and remaining as another race/ethnicity or more than one race/ethnicity. In terms of education, 68.4%, (*n* = 65) participants had completed college or some college, 25.3% had completed high school or GED, and 6.3% attained an advanced degree. Most participants (70.5%, *n* = 67) in the HIV-positive group reported an annual income between $0–25,000. There were 47.9% (*n* = 45) who identified as non-religious. Concerning heath perception, 20.8% of participants reported feeling ‘sick but not terminally ill’ (Table 1).

#### Comparing HIV-positive and self-reported HIV-negative group demographics

There was a considerably higher proportion of male respondents in the HIV-positive group (88.4% vs. 48.3%; *P*<0.001). This difference is consistent with previously established HIV demographics within San Diego County [20] and the United States. The HIV-positive group was older with most participants being between 45–64 (56.3% vs. 22.5%; *P*<0.001). The HIV-negative participants were more likely to have advanced degrees compared with the HIV-positive group (23.5% vs. 6.3%; *P*<0.001). Annual income was significantly lower in the HIV-positive group with 70.5% earning ≤$25,000, compared with 70.0% of HIV-negatives earning >$25,000 (*P*<0.001). There was no significant difference in the number of children between the two groups (*P* = 0.085). Notably, HIV-positive participants were more likely than HIV-negatives to report ‘feeling sick but not terminally-ill’ (20.8% vs. 7.8%; *P* = 0.001) when asked about health status.

### Willingness to participate in research at the EOL

The overwhelming majority of HIV-negative and HIV-positive respondents reported that they would participate in various types of research studies if they were terminally ill (Table 2). Specifically, 90.8% (*n* = 316) of HIV-negative and 82.3% (*n* = 79) of HIV-positive participants demonstrated willingness to participate in EOL research; 95.1% (*n* = 331) of HIV-negative respondents would participate to help a friend or a relative; while 89.6% (*n* = 86) of HIV-positive individuals would participate to help find a cure for HIV/AIDS. Finally, 71.3% (*n* = 67) of HIV-positive and 41.3% (*n* = 145) of HIV-negative respondents would participate in research that involved receiving an experimental and likely high-risk intervention to help find a cure for HIV/AIDS, if they had less than six months to live.

**Table 2:** Attitudes toward research participation by HIV status.

	*Research Participation Attitudes* *n (%)*	
	*HIV-* *(n = 377)*	*HIV+* *(n = 96)*
Willing to participate in research if terminally ill	316 (90.8)	79 (82.3)
If terminally ill, willing to participate in research that would help		
find a cure for HIV/AIDS		86 (89.6)
a friend or relative, but not self	331 (95.1)	
Willing to participate in hazardous-intervention research if it might help find a cure for HIV/AIDS	145 (41.3)	67 (71.3)
Willing to endure		
Blood Draws	297 (78.8)	81 (84.4)
Diarrhea	189 (50.1)	41 (42.7)
Nausea	171 (45.4)	39 (40.6)
Vomiting	108 (28.6)	25 (26.0)
Intramuscular Injection	182 (48.3)	38 (39.6)
Intravenous Injection	210 (55.7)	47 (49.0)
Headache	220 (58.4)	39 (40.6)
Fever	138 (36.6)	33 (34.4)
Willing to shorten lifespan by		
< = 4 weeks	194 (51.4)	38 (39.6)
>4 weeks	67 (17.8)	30 (31.2)
Unwilling to shorten lifespan	116 (30.8)	28 (29.2)
Willing to donate organs to research after death	299 (86.2)	71 (75.5)
Willing to risk exposure to		
Strep Throat	118 (33.6)	
Malaria	79 (22.5)	
Hepatitis C	99 (28.2)	
HIV	105 (29.9)	

Respondents reported varying degrees of willingness to endure certain discomforts as part of a research study: 84.4% (*n* = 81) of HIV-positive and 78.8% (*n* = 297) of HIV-negative participants would endure blood draws; 42.7% (*n* = 41) of HIV-positive and 50.1% (*n* = 189) of HIV-negative participants would endure mild diarrhea; 40.6% (*n* = 39) of HIV-positive and 45.4% (*n* = 171) of HIV-negative participants would endure mild nausea; 26.0% (*n* = 25) of HIV-positive and 28.6% (*n* = 108) of HIV-negative participants would endure vomiting; 39.6% (*n* = 38) of HIV-positive and 48.3% (*n* = 182) of HIV-negative participants would endure intramuscular injections; 49.0% (*n* = 47) of HIV-positive and 55.7% (*n* = 210) of HIV-negative participants would endure intravenous injections; 40.6% (*n* = 39) of HIV-positive and 58.4% (*n* = 220) of HIV-negative participants would endure mild headaches; and 34.4% (*n* = 33) of HIV-positive and 36.6% (*n* = 138) of HIV-negative participants would endure fever (Table 2).

**Fig 1:**
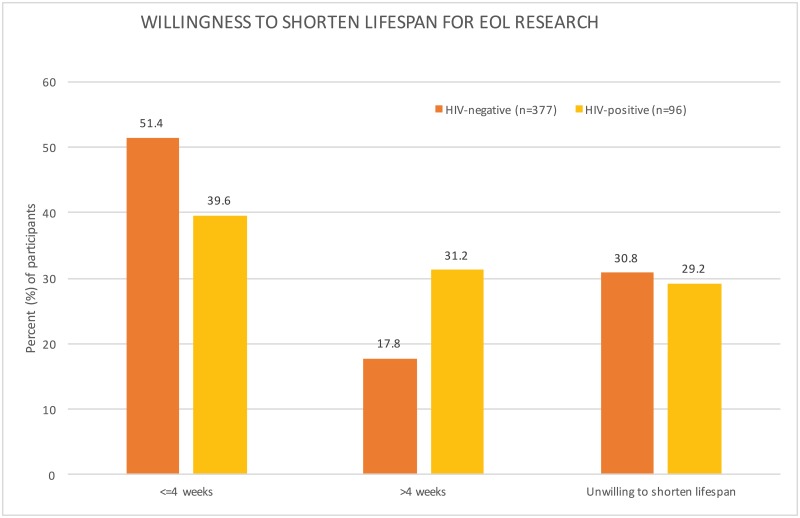
Willingness to shorten lifespan for end of life research. Our surveys found that 69.2% (*n* = 68) of HIV-positive and 70.8% (*n* = 261) of HIV-negative participants were willing to shorten their lifespans for the sake of end of life research. A higher proportion (31.2%) of the HIV-positive group was willing to donate >4 weeks of their lives. About a third of respondents in both groups stated that they were unwilling to shorten their lifespans for the sake of research.

Interestingly, a majority of respondents were willing to shorten their lives by participating in research that would help advance HIV cure research and to donate their organs upon death. Specifically, 39.6% (*n* = 38) of HIV-positive and 51.4% (*n* = 194) of HIV-negative respondents would be willing to decrease their lifespans by up to 4 weeks and 31.2% (*n* = 30) of HIV-positive and 17.8% (*n* = 67) of HIV-negative respondents would decrease their lives by more than 4 weeks (Fig 1). Furthermore, 75.5% (*n* = 71) of HIV-positive and 86.2% (*n* = 299) of HIV-negative participants reported that they would be willing to donate parts of their body to help advance science.

**Fig 2:**
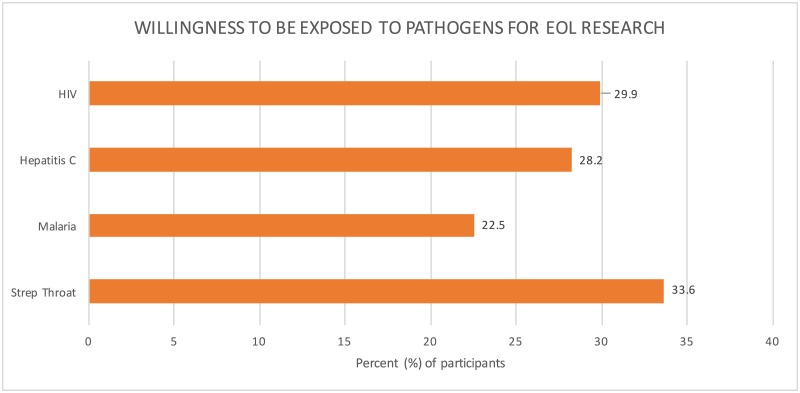
Willingness to be exposed to infectious pathogens for end of life research. Our surveys found that around one-third of the HIV-negative group was willing to be exposed to and infected by an infectious pathogen for the sake of research. Participants were most willing to be exposed to streptococcus (33.6%. *n* = 118) infections, followed by HIV (29.9%, *n* = 105).

Finally, many HIV-negative respondents reported that they would be willing to risk exposure to an infectious agent for the sake of research. Specifically, 33.6% (*n* = 118) would risk exposure to strep throat, 22.5% (*n* = 79) to malaria, 28.2% to Hepatitis C (*n* = 99), and 29.9% (*n* = 105) to HIV to help advance infectious disease research (Fig 2).

## Discussion

Biomedical research is available at the EOL, but mostly for the purposes of alleviating suffering, gaining more time, or trying last ditch interventions to cure illnesses [21,22]. Such studies are mostly conducted in the setting of oncology [23]; these studies are crucial and should not be replaced by other research efforts. There are people, however who do not qualify for, want, or have the opportunity for research in this setting. Instead, when faced with their approaching mortality, such individuals may be willing to participate in research that offers no hope for their condition but that provides one final gift to society. To explore this, we conducted two surveys aimed at understanding hypothetical willingness to participate in clinical research in the last few months of life, aimed at both the general population and separately at the population living with HIV, as a representative of a special population with an incurable chronic infectious disease.

This study provided important observations. First, both HIV-positive and self-reported HIV-negative individuals expressed considerable willingness to participate in infectious disease clinical research at the EOL, even if this research had no relevance to their terminal condition (both >80%). Second, there was a high rate of willingness to donate their body parts for research purposes after death (both >75%). Third, respondents in both groups were hypothetically willing to participate in such research, even if it involved physical discomforts (>70% were willing to endure blood draws and >60% were willing to endure intravenous injections), or even if it reduced their lifespans (both > 50%). Interestingly, HIV-positive participants were more likely to be willing to sacrifice >4 weeks of life to research, compared to the self-reported HIV-negative group. Similarly, another recent study found that HIV-infected people who perceived themselves as ‘not very healthy/not at all healthy’ were significantly more likely to be willing to participate to HIV cure research compared to otherwise healthy chronically HIV-infected individuals, suggesting that people might be more likely to participate when they become (or perceive themselves as) sick [24]. Finally, a high percentage of HIV-negative individuals expressed a willingness to be infected by disease pathogens in the setting of EOL research (i.e. >30% of respondents would allow exposure to HIV, HCV, malaria and/or strep throat). While this last point was provocative, it demonstrates a generally positive attitude and willingness to endure some level of discomfort for altruistic reasons. All of these factors were influenced by various socio-demographic factors, but there were no clear indicators that certain groups would not uniformly participate in the hypothetical infectious disease clinical research at the EOL.

In particular, HIV cure-related research with individuals who are terminally ill could represents a new scientific area, which has the potential to significantly advance the field. For example, by collecting tissue samples from multiple organs at the time of death, we will be able to understand how the virus distributes within the human body and how these tissue reservoirs correlate with each other and with blood reservoirs before death. It also provides an opportunity to understand ethical issues associated with this type of research. As with any clinical research, scientists are expected to obey basic ethical principles, such as beneficence (doing good), non-maleficence (minimizing or preventing harm), autonomy (ensuring informed consent free of coercion), and justice (ensuring the fair distribution of risks and benefits), as articulated by Beauchamp and Childress [25][26]. Additional ethical principles include social value and scientific validity of research, independent review of research, and respect for potential and enrolled participants [27][28].

Currently, there are very few opportunities for terminally-ill people to participate in clinical research, due to various cultural taboos and ethical concerns, such as vulnerability, coercion and exploitation [29]. Vulnerability refers to “increased potential that one’s interests cannot be protected” [30]. Authors have argued against categorizing people at the EOL as being inherently vulnerable [30], and favor describing special protective measures that should be in place given the reality of each clinical study protocol [31]. Coercion is a relational concept that refers to a “credible and irresistible force exerted by one person that negatively limits the option of another person” [30]. Coercion undermines autonomy and can be avoided by clearly demarcating clinical research from medical care at the EOL [32]. Exploitation is an “unfair distribution of the benefits and burdens from a transaction” and must be addressed at the level of the institutional review board overseeing the clinical research [30].

Some may argue that it is unethical to engage people at the EOL in clinical trials [2,33]. However, others feel, along with the principle of representational and distributive justice, that it would be unethical and unjust to withhold this opportunity from them, as long as they have the capability for consent [34]. Informed consent is determined by three fundamental conditions: intentionality, clear understanding and lack of controlling influence [35]. When associated with HIV cure-related research, informed consent must be robust and clearly state that participants will not be cured from HIV from participating in research, but are contributing to creating generalizable knowledge that will benefit future patients and society [28][36][37][38].

Recruiting people for cure studies has been a subject of significant controversy for years [12,15,39,40]. Critics charge that a “conflict of interest” will always exist between the researcher-physician wanting to make scientific strides and the patient-participant hoping for a miraculous treatment or cure [12,15,39,40]. The main critique, which is one of great import, is that this conflict will diminish a participant’s ability to give true informed consent. Prior studies have in fact shown that despite informed consent, participants in these clinical trials hold on to hope for therapeutic benefit, a phenomenon coined “therapeutic optimism” in the literature [41–43]. Other scholars disagree with this claim, citing that hope does not, in itself, compromise informed consent [44]. We would argue that EOL studies are actually if anything devoid of the risk for “therapeutic optimism”, as they do not offer any hope of clinical improvement. They do allow interested parties however the choice of participating in a study that could improve the life of another, and in that, provide a Last Gift to the society. In this sense, participants must understand that they enter into a ‘gifting relationship’ with the research team, future patients and the HIV cure research community [16].

Denying individuals the opportunity to participate in research at the EOL based on a general assumption of vulnerability is simplistic. It undermines their autonomy and right to self-determination and does not take the diversity of the population into account [34]. A recent review concerning EOL research and its impact on participants, demonstrated that the ethical concerns regarding research participation at EOL are often unjustified [34]. Of course, these studies require careful design and execution that incorporates sensitivity to participants’ needs, concerns and preferences, as well as observance of all legal requirements [45][46][47]. The research that an individual engages in should be tailored ethically and specifically to that participant’s illness and expectations [45]. Interestingly, health professionals have been found to be the most likely to raise concerns regarding involving participants at the EOL in research, while patients themselves were generally willing to contribute to research and reported this engagement as a positive experience [34].

Another argument against research of this nature could be that terminally ill individuals have traditionally been hard to recruit and retain in studies aimed to better their current condition [30,32,48–50]. However, and as our study shows, with 95.1% of HIV-negative respondents willing to participate in EOL research to help a friend or relative, and 89.6% of those with HIV to help find a cure for HIV/AIDS, altruism may be a better motivator than self-gain for those approaching the EOL [51]. Altruism could outweigh the discomfort or uneasiness that participating in a clinical trial may bring [52]. Another instance is a mother with muscular dystrophy dying of cancer who wants to participate in a clinical trial on muscular dystrophy to possibly improve the quality of life for her son, who also has the disease. Similarly, a person living with HIV might be willing to participate to HIV-research at the EOL to help his/her infected partner or peers. This is analogous to the early HIV epidemic when thousands of gay men and other affected community members enrolled in research at enormous self-sacrifice, with little or no personal benefit [53]. Those same altruistic individuals have often been denied (for various reasons) the opportunity to contribute to HIV research in recent decades.

There will be various obstacles that this type of research will encounter, but we argue that these barriers can be overcome by understanding and addressing the wants and needs of the participant population in due course.

This study has a number of limitations. First and foremost, the survey information presented in this study was not collected from people at their EOL, but rather from two convenience groups recruited through MTurk and our local HIV clinic in San Diego. People may feel very differently about research participation when they actually have a terminal illness with only six months left to live and our results may not be generalizable across larger populations. Self-report might not be predictive of future behavior. However, websites (as MTurk) are widely used in behavioral research and offer a useful platform that provides convenient access to a large set of people who are willing to undertake tasks at a relatively low cost [54]. We also previously performed unstructured interviews of 12 people receiving hospice services. All of these individuals expressed a desire to be able to ‘*give back’* in some way, especially at this time near the EOL [17]. Further, the sample size was relatively small which might have limited our ability to perform stratified analysis. Another key limitation is that the differences between recruitment methods, groups and sample sizes precluded statistical comparisons between people living with HIV and people not living with HIV and the paper mostly focused on descriptive data. Lastly, generalizability of findings from research at EOL might not be directly applicable to the general population (e.g. efficacy of strategies to cure HIV might be very different at the EOL compared to otherwise healthy HIV-infected people).

We call for a broad, frank, and pragmatic discussion about research near the EOL, which may represent a new, innovative paradigm in how we conduct research with human participants (“less to lose” versus “otherwise healthy volunteers”). In this dialogue, we envision that cultural, ethical and legal challenges can be resolved, and that research safeguards can be developed. Research in this setting offers a valuable alternative to animal testing which is more generalizable to the human condition, and allows those who are dying one more chance to give to future generations. We must tackle the cultural, ethical and infrastructure barriers that prevent people from participating in clinical research at the EOL to help overcome many of the important health challenges of our day. To address this issues, we propose the following plan of action: 1) Perform a similar survey among people who are at the EOL; 2) Consider all ethical aspects for research at the EOL (in particular the role of informed consent, autonomy, altruism and vulnerability in the decision making process); 3) Recognize the role of family members and other stakeholders in decisions to participate in research at the EOL; 4) Consider all practical aspects of research at the EOL so that it can be done effectively and ethically; 5) Take into account values regarding quantity versus quality of life at each step of this process.
